# The role of body mass index and diabetes in the development of acute organ failure and subsequent mortality in an observational cohort

**DOI:** 10.1186/cc5051

**Published:** 2006-09-25

**Authors:** Katarina Slynkova, David M Mannino, Greg S Martin, Richard S Morehead, Dennis E Doherty

**Affiliations:** 1 Division of Pulmonary and Critical Care Medicine, University of Kentucky Medical Center, and Veteran's Administration Medical Center, 740 South Limestone, K 528 Kentucky Clinic, Lexington, KY 40536, USA; 2 Division of Pulmonary, Allergy, and Critical Care, Emory University School of Medicine, 49 Jesse Hill Jr Dr SE, Atlanta, GA 30303, USA

## Abstract

**Introduction:**

Several studies have shown a correlation between body mass index (BMI) and both the development of critical illness and adverse outcomes in critically ill patients. The goal of our study was to examine this relationship prospectively with particular attention to the influence of concomitant diabetes mellitus (DM).

**Methods:**

We analyzed data from 15,408 participants in the Atherosclerosis Risk in Communities (ARIC) study for this analysis. BMI and the presence of DM were defined at baseline. We defined 'acute organ failure' as those subjects who met a standard definition with diagnostic codes abstracted from hospitalization records. Outcomes assessed included the following: risk of the development of acute organ failure within three years of the baseline examination; in-hospital death while ill with acute organ failure; and death at three years among all subjects and among those with acute organ failure.

**Results:**

At baseline, participants with a BMI of at least 30 were more likely than those in lower BMI categories to have DM (22.4% versus 7.9%, *p *< 0.01). Overall, BMI was not a significant predictor of developing acute organ failure. The risk for developing acute organ failure was increased among subjects with DM in comparison with those without DM (2.4% versus 0.7%, *p *< 0.01). Among subjects with organ failure, both in-hospital mortality (46.5% versus 12.2%, *p *< 0.01) and 3-year mortality (51.2% versus 21.1%, *p *< 0.01) was higher in subjects with DM.

**Conclusion:**

Our findings suggest that obesity by itself is not a significant predictor of either acute organ failure or death during or after acute organ failure in this cohort. However, the presence of DM, which is related to obesity, is a strong predictor of both acute organ failure and death after acute organ failure.

## Introduction

Obesity is one of the major health problems in our society and its prevalence is rising worldwide [1,2]. Currently, about 130 million US adults, 65% of the population, are overweight or obese. In addition, an increasing proportion of children in the USA are either overweight or obese, and this number has almost doubled over the past two decades [1].

Excess body weight increases the risk of hypertension, coronary artery disease, stroke, sleep apnea, and certain cancers [3-6]. Several population studies have described an association between body mass index (BMI) and mortality as a U-shaped curve, demonstrating increased mortality in the lowest and highest BMI distribution, even when controlling for age, smoking, and history of other comorbidities [7-11]. Obesity is also strongly associated with an increased risk of diabetes [12].

However, the influence of BMI on morbidity and mortality in critically ill patients is still controversial [13,14]. Retrospective analysis of 63,646 patients from a multi-institutional intensive care unit database showed increased mortality in underweight patients (BMI ≤ 20) but not in overweight or obese patients [15].

By contrast, Goulenok and colleagues demonstrated, in a prospective study of critically ill patients, that a higher BMI was associated with increased mortality [16]. The authors did not include diabetes as an independent variable in the analysis and could not explain the results even though the infection rate and the duration of mechanical ventilation were not increased in the obese group. Studies of critically ill patients who are morbidly obese (BMI > 40) have shown more complications and higher mortality but have not controlled for diabetes mellitus (DM) [17,18].

The relationship between obesity and diabetes, and particularly the modifying effect of the latter, in the development of acute organ failure, critical illness, and subsequent mortality is not well defined. A better understanding of this relationship may help to improve interventions and therefore outcomes in this commonly seen group of patients.

The goals of this analysis were to examine the effect of BMI in the development of acute organ failure and subsequent mortality in a large, population-based, prospective cohort. We also wished to determine the role of DM in modifying the effect of obesity.

## Materials and methods

### Study population

The Atherosclerosis Risk in Communities (ARIC) study is a prospective, population-based study of the natural history and etiology of cardiovascular disease. The ARIC population is a probability sample of 15,792 men and women aged from 44 to 66 years from four US communities (Suburban Minneapolis, Minnesota; Washington County, Maryland; Forsyth County, North Carolina, and Jackson, Mississippi) originally studied in 1986 to 1989 [19]. The cohort population reflects the demographic sample of the community except Jackson, where only African-Americans were included. Participants underwent baseline clinical examination, BMI measurement, and pulmonary function testing, and provided information on their medical and smoking history and educational background. The study was approved by institutional review boards at the clinical sites, and informed consent was obtained from all participants.

### Subjects

We restricted this analysis to participants for whom there was complete baseline data on variables important to this analysis, including BMI, DM status, lung function testing, and smoking history. We excluded 208 subjects for whom there were missing data on diabetes status, 131 subjects for whom there were missing data on lung function, and 45 subjects for whom there were missing data on other key variables, resulting in 15,408 participants in our analytic cohort.

Hospitalization summaries, including International Classification of Disease, Ninth Revision (ICD-9) codes for the discharge diagnoses and procedures performed, and disposition were available for all study participants. In addition, data from death certificates were also available.

### Definitions of variables

#### Acute organ failure

We identified these subjects as those who were hospitalized and developed acute organ dysfunction as defined by Martin and colleagues [20] using ICD-9 codes (sepsis was not required as part of this definition). Diagnostic codes for organ dysfunction in these systems were included: respiratory, cardiovascular, renal, hepatic, hematologic, metabolic, and neurologic. Although one would assume that most inpatients who develop 'acute organ failure' would be critically ill and admitted to an intensive care unit, the site of in-hospital treatment was not available in the database.

#### Body mass index

Weight and height were measured, and BMI, calculated as weight (in kilograms) divided by the square of the height (in meters) was defined at baseline. Each group was further divided into four BMI subgroups, as follows: underweight (defined as BMI ≤ 20 kg/m^2^), normal (defined as a BMI of 21 to 24 kg/m^2^), overweight (defined as a BMI of 25 to 29 kg/m^2^) and obese (defined as BMI ≥ 30 kg/m^2^). These are commonly accepted standard subgroups except that we did adjust the underweight group, generally defined as BMI ≤ 18.5, to increase the sample size in this group and provide more stable estimates [1].

#### Diabetes status

We classified subjects as having DM at the baseline examination if they had any of the following: a positive response to the question 'Has a doctor ever told you that you had diabetes (sugar in the blood)?'; a report of taking medication for 'diabetes or high blood sugar' in the two weeks before the survey; a fasting blood sugar of 126 or higher. We were not able to distinguish between types 1 and 2 DM.

#### Lung function

We used a modification of the criteria developed by the Global Initiative for Chronic Obstructive Lung Disease (GOLD) [21] to classify subjects according to their GOLD stages of chronic obstructive pulmonary disease (COPD): GOLD stage 3 or 4 (forced expiratory volume in 1 second (FEV_1_)/forced vital capacity (FVC) < 0.70 and FEV_1 _< 50% predicted), GOLD stage 2 (FEV_1_/FVC < 0.70 and FEV_1 _≥ 50 to < 80% predicted), GOLD stage 1 (FEV_1_/FVC < 0.70 and FEV_1 _≥ 80%), restricted (FEV_1_/FVC ≥ 0.70 and FVC < 80% predicted), GOLD stage 0 (presence of respiratory symptoms in the absence of any lung function abnormality), and no lung disease. Bronchodilator response was not evaluated in this survey, so the classification is based on the 'pre-bronchodilator' level. We used data from asymptomatic never-smokers with no lung disease, stratified by race and sex, to develop internal prediction equations to determine normal values for lung function.

#### Other variables

Respondents with positive responses to the questions 'Have you ever smoked cigarettes?' and 'Do you now smoke cigarettes?' were classified as 'ever smokers' and 'current smokers', respectively. Education level was categorized as less than high school, completion of high school, or more than high school. Baseline age was grouped into four strata: 44 to 49 years, 50 to 54 years, 55 to 59 years, and 60 to 66 years.

### Outcome variables

The primary outcomes of interest were the risk of the development of acute organ failure within three years of the baseline examination, in-hospital mortality related to any acute organ failure during that period, and all-cause mortality at three years.

### Analysis

All analyses were conducted with SAS version 8.2 (SAS Institute, Cary, NC, USA), SUDAAN version 8.0 (RTI, Research Triangle Park, NC, USA) and SPSS version 10 (SPSS Inc, Chicago, IL, USA). Multivariable logistic regression models were developed for the outcomes of any hospitalization with acute organ failure, death at three years, and in-hospital death among those with acute organ failure. Models were adjusted for age, sex, race, smoking status, education level, BMI, diabetes status, and lung function status.

Cox proportional hazard regression models were developed with the SUDAAN procedure SURVIVAL to account for differential follow-up in ARIC participants. Time of follow-up was used as the underlying time metric. For deaths, the exit date was the date of death reported on the death certificate and, for survivors, the exit date was the date on which the participant was last known to be alive. Plots of the log-log survival curves for each covariate were used to show that the proportional-hazards assumptions were met. Age, sex, race, smoking status, education level, BMI, diabetes status, and lung function status were included in the adjusted models, and the models were evaluated for interactions.

## Results

A total of 15,792 adults aged 44 to 66 years participated in the ARIC study. We excluded 384 subjects with missing data either on diabetes status, lung function or other key variables, resulting in 15,408 subjects in our analytic cohort.

The demographic characteristics of the cohort population at baseline and their outcomes are shown in Table 1. About one-third of the study population (29.9%) had an ideal body weight (BMI 21 to 24), 39.3% were overweight (BMI 25 to 29) and 27.5% were obese (BMI ≥ 30). This distribution reflects the US population well, with an estimated prevalence of about 30% obese and 35% overweight [1]. The mean BMIs of the cohort by BMI category was 18.9 (BMI < 20), 22.9 (BMI = 20 to 24), 27.3 (BMI = 25 to 29), and 34.5 (BMI ≥ 30).

Overall, 1,830 (11.9%) of all participants had DM. Participants with DM were more likely than those without DM to have a BMI of 30 or more (52% versus 24%, *p *< 0.01). The prevalence of DM increased with increasing BMI: 4.4% for BMI < 20, 4.9% for BMI = 20 to 24, 10% for BMI = 25 to 29, and 22.4% for BMI ≥ 30. Of the 1,830 subjects classified as having DM, 636 (43%) were classified solely on the basis of a fasting blood sugar of more than 126 mg/dl.

The development of acute organ failure among participants with different BMI subgroups was almost identical: 0.9% of subjects with ideal body weight developed acute organ failure, in comparison with 0.8% and 0.9% for overweight and obese subjects, respectively. BMI subgroups had similar mortality at the 3-year follow-up (Table 1). Within three years 5.4% of subjects with diabetes, in contrast with 1.6% of subjects without diabetes, died (Table 1).

Risk factors for developing acute organ failure within three years of the baseline evaluation and death within three years are displayed in Table 2. The significant risk factors for acute organ failure included older age, male sex, DM, and lower levels of lung function. These same factors, along with smoking, black race, and a lower educational level, predicted death within three years. The presence of DM was among the strongest independent predictors for the risk of acute organ failure, with a threefold increased risk (odds ratio 3.2; 95% confidence interval 2.1 to 4.7), and for all-cause mortality at 3 years (odds ratio 2.7; 95% confidence interval 2.1 to 3.5).

Table 3 displays the risk of the development of acute organ failure, in-hospital mortality among those with acute organ failure, and mortality at three years among those with acute organ failure by DM status. Subjects with DM were both more likely to develop a organ failure (2.4% versus 0.7%, *p *< 0.01) and to die during that hospitalization (46.5% versus 12.2%, *p *< 0.01) than were subjects without DM.

**Figure 1:**
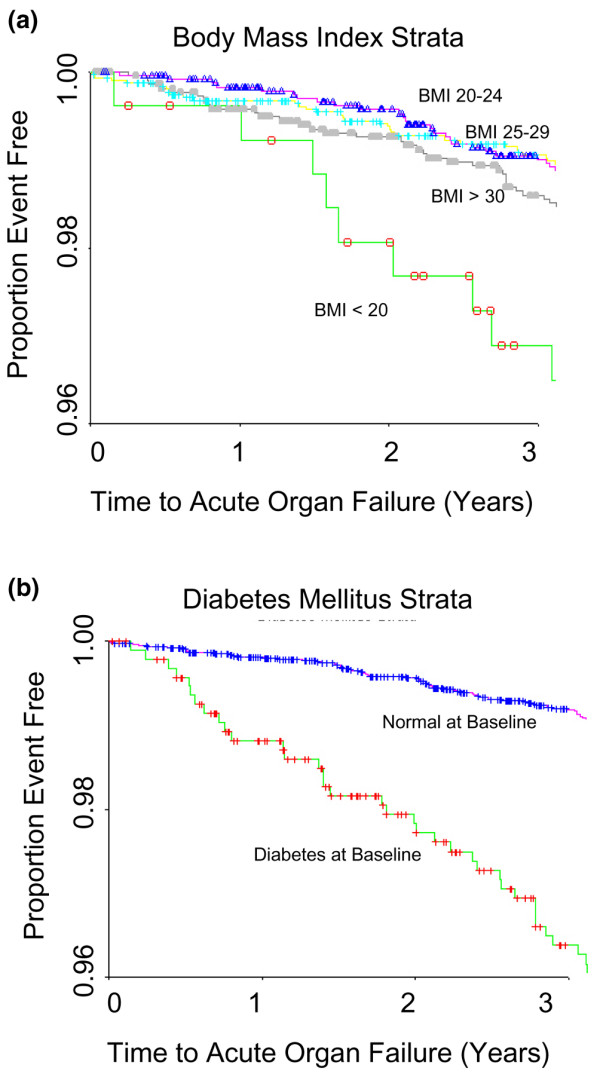
Time to development of acute organ failure among study participants. The results are stratified by body mass index **(a) **and diabetes mellitus **(b)**. From the Atherosclerosis Risk in Communities study 1986–1989 and follow-up.

Time to the development of acute organ failure is presented in Figure 1, stratified by BMI (Figure 1a) and by DM status (Figure 1b). Cox proportional-hazards models in Table 4 show that in comparison with subjects without DM with BMIs of 21 to 24, subjects with DM had about a threefold higher risk of developing acute organ failure. Although the confidence intervals were wide because of sample size, a similar trend for a higher risk of in-hospital mortality was also noted among subjects with DM.

## Discussion

The results of this large prospective cohort study of middle-aged adults in the USA suggest that the development of acute organ failure and death after acute organ failure is more related to the presence of DM than to an increased BMI. Our definition of acute organ failure is, we believe, a surrogate for critical illness, in that these events occurred in the setting of a hospitalization. Our results do not support the contention that obesity itself is a risk factor for increased mortality in patients with acute organ failure. It brings up a new perspective on this still controversial subject of obesity, critical illness, and mortality. In addition, our findings did not confirm increased mortality in overweight or obese critically ill patients without DM.

Previous studies have shown that a low BMI is a significant predictor of higher hospital mortality [9,11]. This increased risk among patients with low BMIs has also been demonstrated in critically ill patients, although these data are limited [15,22]. Our data suggested that subjects with low BMI developed organ failure earlier (Figure 1 and Table 4) and had a higher risk of in-hospital death, although the confidence intervals were wide and did not reach significance.

Conversely, other epidemiological studies have shown that high BMI is associated with higher all-cause mortality rates [8,9]. In one large prospective cohort study the mortality rate for overweight non-smoking men was 3.9 times higher then for non-smoking men of ideal weight [9]. Some authors have postulated that obesity may not be a risk factor with regard to subsequent mortality in critically ill individuals, but rather that it could have a beneficial role of 'nutritional reserve' in the face of critical illness [15].

The relationship between an increased BMI and DM has been well established [12,23,24]. Our data, similarly, showed that subjects with BMIs ≥ 30 had a fourfold higher prevalence of DM than subjects with BMIs of 20 to 24. Weight gain, which frequently precedes the onset of diabetes, is also considered to be a risk factor for this disease [25,26]. Both increased insulin secretion and insulin resistance result from obesity, and hyperglycemia and insulin resistance are the hallmarks of diabetes. Hyperglycemia has been shown to increase the release of pro-inflammatory mediators such as IL-6, IL-8, and TNF, which are important in inflammation [27]. In addition, high glucose levels have been shown to have deleterious effects on optimal macrophage and neutrophil function [28]. These negative effects of hyperglycemia are responsible for the increased risk of infection, cardiovascular diseases, organ dysfunction, and certain cancers, as has been shown recently [29-31].

Our findings suggest that DM and associated hyperglycemia with insulin resistance, rather than obesity itself, is responsible for the development of acute organ failure and subsequent adverse outcomes in this middle-aged US population. This hypothesis can be further supported by recent and consistent evidence suggesting that the outcomes in critically ill patients are improved by both tight glucose control and insulin therapy with its anti-inflammatory properties [32-34].

This study has several limitations. First, the accuracy of ICD-9-CM (the clinical modification of ICD-9) coding in identifying specific medical conditions is unproven [35], yet it is increasingly used for epidemiologic purposes [20]. In critical illness, the sensitivity and specificity of the diagnostic code for sepsis were estimated to be 87.7% and 98.8%, respectively. However, the use of ICD-9 codes for epidemiologic estimates may underestimate true incidence [36].

Second, we have focused on patients who were hospitalized and developed acute organ dysfunction, although we acknowledge that definitions in critical care population are not well validated. For example, some patients who had chronic organ failure might have been captured with our definition, and others might have developed organ failure and died without being hospitalized.

Third, we did not subcategorize patients who were morbidly obese (BMI > 40) because of the small sample size (about 3%). At the time of the study, morbid obesity was not as significant problem as it has become over the past 15 years. It seems that increased mortality and increased risk of multiple complications in a morbidly obese population are more obvious, as shown in several retrospective studies, and should therefore be studied separately [14,17,18,37].

Fourth, patients were enrolled in this trial in 1986 to 1989, and both treatment for diabetes and treatment of acute organ failure may have changed in the years since then. However, it is noteworthy that is that in the USA the proportion of diabetic patients who are 'well controlled', on the basis of hemoglobin A1C levels, has not improved over the past two decades [38].

Fifth, our definition of DM might not have included all subjects with this disease, in that we did not perform glucose tolerance testing or other confirmatory testing. However, this potential misclassification would have biased our results towards not finding an effect in the DM population.

Finally, we included only three years of follow-up in this analysis. This was done to focus on the short-term risk of BMI and DM on acute organ failure. Over the long term, patients with high BMIs are more likely to develop DM and would in all likelihood have an increased risk of acute organ failure and critical illness subsequently.

## Conclusion

Results of this study indicate that the presence of DM, rather than an increased BMI, accounts for a higher risk of acute organ failure and associated mortality. These findings call for further investigation to determine the mechanisms that underlie this complex relationship between obesity, diabetes, and critical illness. It will help to optimize care, which will result in improved outcomes and a decrease in the associated health care costs currently being expended in this growing population of patients.

## Key messages

• In this middle-aged cohort, diabetics had a threefold higher incidence of organ failure over three years than non-diabetics.

• After adjusting for diabetes, BMI did not predict organ failure in this cohort.

• Among patients with organ failure in this cohort, diabetics had more than twice the risk of in-hospital death than non-diabetics.

• After adjusting for diabetes, an increased BMI did not predict in-hospital death among patients with organ failure in this cohort.

## Abbreviations

ARIC = Atherosclerosis Risk in Communities; BMI = body mass index; DM = diabetes mellitus; FEV_1 _= forced expiratory volume in 1 second; FVC = forced vital capacity; GOLD = Global Initiative for Chronic Obstructive Lung Disease; ICD-9; International Classification of Disease, Ninth Revision; IL = interleukin; TNF = tumor necrosis factor.

## Competing interests

The authors declare that they have no competing interests.

## Authors' contributions

KS and RSM designed the study, interpreted the data analysis, and drafted the manuscript. DMM and GSM designed the study, performed and interpreted the data analysis, and drafted the manuscript. DED interpreted the data analysis and revised the manuscript. All authors read and approved the final manuscript.

## Figures and Tables

**Table 1:** Demographic distribution and outcomes of study participants

Category	*n *(%)	Acute organ failure within 3 years (%)	Death within 3 years (%)
Age group			
44–49	4,106 (26.7)	0.4	1.1
50–54	4,007 (26.0)	0.6	1.0
55–59	3,761 (24.4)	0.9	2.3
60–66	3,534 (22.9)	1.8	4.0
Sex			
Male	6,918 (44.9)	1.1	2.7
Female	8,490 (55.1)	0.6	1.5
Race			
White	11,365 (73.8)	0.9	1.6
Black	4,043 (26.2)	0.7	3.2
Smoking status			
Current smoker	4,394 (28.5)	1.2	3.4
Former smoker	4,964 (32.2)	0.9	1.8
Never smoker	6,050 (39.3)	0.6	1.3
Diabetes mellitus			
Yes	1,830 (11.9)	2.4	5.4
No	13,578 (88.1)	0.7	1.6
Body mass index			
<20	506 (3.3)	1.4	3.0
20–24	4,610 (29.9)	0.9	2.0
25–29	6,058 (39.3)	0.8	1.9
≥30	4,234 (27.5)	0.9	2.2
Education level			
<12 years	3,646 (23.7)	1.4	3.6
12 years	4,991 (32.4)	0.6	1.9
≥12 years	6,771 (43.9)	0.7	1.3
GOLD category^a^			
GOLD 3 or 4	280 (1.8)	5.4	8.6
GOLD 2	1,469 (9.5)	1.9	4.1
GOLD 1	1,677 (10.9)	0.9	2.0
GOLD 0	2,170 (14.1)	1.2	2.2
Restricted	1,295 (8.4)	1.4	3.9
Normal	8,517 (55.3)	0.4	1.1
Total	15,408	0.9	2.0

Data are taken from the Atherosclerosis Risk in Communities study 1986–1989 and follow-up. FEV_1_, forced expiratory volume in 1 second; FVC, forced vital capacity. ^a^Modified Global Initiative for Chronic Obstructive Lung Disease (GOLD) stage 3 or 4 (FEV_1_/FVC < 0.70 and FEV_1 _< 50% predicted), GOLD stage 2 (FEV_1_/FVC < 0.70 and FEV_1 _≥ 50 to < 80% predicted), GOLD stage 1 (FEV_1_/FVC < 0.70 and FEV_1 _≥ 80%), restricted (FEV_1_/FVC ≥ 0.70 and FVC < 80% predicted), GOLD stage 0 (presence of respiratory symptoms in the absence of any lung function abnormality). All lung function measurements were pre-bronchodilator values.

**Table 2:** Odds ratios, from multivariable logistic regression models, for outcomes within 3 years

Category	*n *(%)	Acute organ failure within 3 years; odds ratio (95% CI)^b^	Death within 3 years; odds ratio (95% CI)
Age group			
44–49	4,106 (26.7)	1.0	1.0
50–54	4,007 (26.0)	1.2 (0.6–2.4)	0.9 (0.6–1.4)
55–59	3,761 (24.4)	1.6 (0.9–3.0)	1.8 (1.2–2.6)
60–66	3,534 (22.9)	2.9 (1.6–5.2)	3.0 (2.1–4.3)
Sex			
Male	6,918 (44.9)	1.0	1.0
Female	8,490 (55.1)	0.6 (0.4–0.9)	0.6 (0.4–0.7)
Race			
White	11,365 (73.8)	1.0	1.0
Black	4,043 (26.2)	0.7 (0.5–1.1)	2.0 (1.5–2.6)
Smoking status			
Current smoker	4,394 (28.5)	1.2 (0.7–1.9)	1.8 (1.4–2.5)
Former smoker	4,964 (32.2)	1.0 (0.6–1.6)	1.1 (0.8–1.5)
Never smoker	6,050 (39.3)	1.0	1.0
Diabetes mellitus			
Yes	1,830 (11.9)	3.2 (2.1–4.7)	2.7 (2.1–3.5)
No	13,578 (88.1)	1.0	1.0
Body mass index			
<20	506 (3.3)	1.3 (0.6–2.8)	1.1 (0.7–2.0)
20–24	4,610 (29.9)	1.0	1.0
25–29	6,058 (39.3)	0.8 (0.5–1.2)	0.8 (0.6–1.1)
≥30	4,234 (27.5)	0.9 (0.6–1.4)	0.8 (0.6–1.1)
Education level			
<12 years	3,646 (23.7)	1.3 (0.8–1.9)	1.5 (1.1–2.0)
12 years	4,991 (32.4)	0.8 (0.5–1.3)	1.4 (1.1–1.9)
≥12 years	6,771 (43.9)	1.0	1.0
GOLD category^a^			
GOLD 3 or 4	280 (1.8)	9.4 (4.8–18.4)	4.3 (2.6–7.0)
GOLD 2	1,469 (9.5)	3.6 (2.0–6.4)	2.2 (1.5–3.2)
GOLD 1	1,677 (10.9)	1.9 (0.98–3.6)	1.3 (0.9–1.9)
GOLD 0	2,170 (14.1)	2.8 (1.6–5.2)	2.4 (1.7–3.5)
Restricted	1,295 (8.4)	2.9 (1.7–5.0)	1.6 (1.1–2.3)
Normal	8,517 (55.3)	1.0	1.0

Data are taken from the Atherosclerosis Risk in Communities study 1986–1989 and follow-up. FEV_1_, forced expiratory volume in 1 second; FVC, forced vital capacity. ^a^Modified Global Initiative for Chronic Obstructive Lung Disease (GOLD) stage 3 or 4 (FEV_1_/FVC < 0.70 and FEV_1 _< 50% predicted), GOLD stage 2 (FEV_1_/FVC < 0.70 and FEV_1 _≥ 50 to < 80% predicted), GOLD stage 1 (FEV_1_/FVC < 0.70 and FEV_1 _≥ 80%), restricted (FEV_1_/FVC ≥ 0.70 and FVC < 80% predicted), GOLD stage 0 (presence of respiratory symptoms in the absence of any lung function abnormality). All lung function measurements were pre-bronchodilator values. ^b^95% confidence intervals (CI) that do not include 1.0 are significant at the *p *= 0.05 level.

**Table 3:** The risk of development of critical illness, in-hospital mortality and all-cause mortality at 3 years

Diabetes mellitus	Number of subjects	Acute organ failure hospitalization	Acute organ failure with in-hospital death	Acute organ failure with all-cause mortality at 3 years
Absent				
BMI ≤ 20	484	7 (1.5)	28.6	28.6
BMI 21–24	4,383	32 (0.7)	12.5	15.6
BMI 25–29	5,424	30 (0.6)	13.3	23.3
BMI ≥ 30	3,287	21 (0.6)	4.8	23.8
Total	13,578	90 (0.7)	12.2	21.1
Present				
BMI ≤ 20	22	0	NA	NA
BMI 21–24	227	8 (3.5)	37.5	37.5
BMI 25–29	634	16 (2.5)	62.5	62.5
BMI ≥ 30	947	19 (2.0)	36.8	47.4
Total	1,830	43 (2.4)	46.5	51.2

Data are taken from from the Atherosclerosis Risk in Communities study 1986–1989 and follow-up, and are presented as proportions (%) over the 3-year study follow-up period. BMI, body mass index.

**Table 4:** Results from models predicting time to acute organ failure and risk of in-hospital death

Diabetes mellitus	Body mass index	Time to acute organ failure, hazard ratio (95% CI)	Risk of in-hospital death during acute organ failure, odds ratio (95% CI)
No	≤20	1.5 (0.7–3.4)	2.3 (0.3–15.3)
No	21–24	1.0	1.0
No	25–29	0.7 (0.4–1.2)	0.7 (0.2–3.2)
No	≥30	0.9 (0.5–1.6)	0.2 (0.04–1.7)
Yes	≤20	NA	NA
Yes	21–24	3.4 (1.6–7.4)	6.8 (1.00–45.8)
Yes	25–29	2.8 (1.5–5.1)	3.2 (0.9–11.9)
Yes	≥30	2.7 (1.5–4.9)	2.1 (0.5–8.6)

Data are taken from the Atherosclerosis Risk in Communities study 1986–1989 and follow-up. Models were adjusted for age, sex, race, smoking status, education level, body mass index, diabetes status, and lung function status. 95% confidence intervals (CI) that do not include 1.0 are significant at the *p *= 0.05 level. NA, not available (there were no critical illness hospitalizations in this subgroup).
